# Editorial: Molecular Mechanisms Involved in Heart Failure, Parkinson’s, and Alzheimer’s Diseases

**DOI:** 10.3389/fmolb.2021.754987

**Published:** 2021-09-08

**Authors:** Grazia Daniela Femminella, Gennaro Pagano, Daniela Liccardo, Alessandro Cannavo

**Affiliations:** ^1^ Department of Translational Medical Science, Federico II University of Naples, Naples, Italy; ^2^ Roche Pharmaceutical Research and Early Development, NRD Neuroscience and Rare Diseases, Roche Innovation Center, F. Hoffmann-La Roche Ltd, Basel, Switzerland; ^3^ Center for Translational Medical Science, Lewis Katz School of Medicine, Temple University, Philadelphia, PA, United States

**Keywords:** heart failure, Alzheheimer’s disease, mechanism, pathogenesis, signaling

As life expectancy increases, chronic degenerative disorders such as heart failure (HF) and Alzheimer’s diseases are a growing pandemic for older adults. No available treatment can slow the progression of these diseases, and massive increases in health care costs are predicted in the coming years. Understanding the molecular mechanisms involved in the pathogenesis of such disorders would provide the basis for identifying druggable targets and facilitating novel treatments to prevent a decline in functional activities of daily living hence improving the quality of life of older adults.

In this Research Topic, we provided updates on the most advanced knowledge on the interlinks between molecular triggering factors and signaling pathways and how they may influence almost every aspect of cardiovascular, neurological, and other chronic degenerative diseases.

For instance, in their Review article, Gariballa and Ali explored the role of endoplasmic reticulum (ER) quality control mechanisms behind the pathogenesis of genetic diseases associated with alterations in the components of the TGFβ signaling pathway. As emerged in their analysis, the authors found that about 47 monogenic diseases are associated with genetic mutations in 24 out of 41 TGFβ components. The authors emphasized the urgency of establishing novel approaches in modulating the molecular pathway of mutant TGFβ components restoring their protein folding and trafficking as the final therapeutic goal. Importantly, protein misfolding is a well-recognized pathogenic mechanism involved in several disorders, and novel strategies to prevent such abnormal processes are needed. Therefore, the authors propose genetic manipulation of ER-associated protein degradation (ERAD) network to enhance mutant protein folding, localization, and activity as a novel strategy for preserving biologically functional properties of the TGFβ signaling pathway, counteracting the development of several chronic-degenerative disorders.

Further, in their interesting analysis, Chen et al. described the pathogenetic role of protein-misfolding in HF. Notably, these authors discussed the vital role of mitochondrial chaperones and proteases in the perturbation of protein homeostasis showing the mechanisms by which these influence cardiomyocyte functionality and survival.

Importantly, alteration in protein folding has been recognized as a process leading to neurodegeneration and Alzheimer’s disease (AD) development (Uddin et al., 2021). Indeed, the extracellular deposition of aggregated beta-amyloid peptides results in neuronal cell dysfunction and apoptosis (Liccardo et al., 2020). Moreover, several previous findings demonstrated how β-amyloid (Aβ) accumulation activates microglia cells, the resident immune cellular population of the central nervous system (CNS), initiating the chronic inflammatory response which participates in the neurodegeneration process (Femminella et al., 2018; Liccardo et al., 2020). The importance of microglia has been supported by several pre-clinical and clinical reports and by Genome-Wide Association Studies (GWAS), showing that AD-risk single-nucleotide polymorphisms are highly expressed in microglia (Griciuc and Tanzi, 2021).

GWAS are fundamental for identifying loci associated with diseases, although they often do not point to causal polymorphisms. In this sense, Kretzschmar et al. replicated in the South Brazilian samples some of the main associations reported in late-onset AD (LOAD)-GWAS performed in European populations. They investigated the potential functional role of these variants in LOAD development. Notably, of 18 single-nucleotide polymorphisms (SNPs) investigated, only four were associated in the population analyzed. Moreover, these authors found that six lncRNAs are possibly playing a role in LOAD.

Notably, among the polymorphic variants identified by GWAS, those of the gene encoding for triggering receptor expressed on myeloid cells 2 (TREM2) have been found highly associated with the risk of developing AD (Gratuze et al., 2018; Griciuc and Tanzi, 2021). Indeed, alteration in the expression activity of TREM2 contributes to shifting the microglia phenotype into a neurodegenerative pattern, increased Aβ aggregation, and decreased degradation. Herein, in their original article Ferri et al. aimed at studying how the concentrations of soluble TREM2 in the cerebrospinal fluid (CSF) of AD patients correlate with the concentrations of other CSF markers of AD progression. Of note, these authors observed a positive association between sTREM2 and phosphorylated Tau concentrations and between plasmatic levels of sTREM2 and the levels of Aβ1−42 in the CSF.

Neuroinflammation has been further evaluated in the perspective article by Kretzschmar et al. who reviewed the current evidence on the role of the complement system in AD. These authors explored the possible involvement of the complement system in the recruitment of neutrophils and the formation of neutrophil extracellular traps (NETs). NETs are involved in inflammation associated with autoimmune conditions and have been observed adjacent to amyloid plaques in the brains of both animals and patients with AD. Moreover, this study reports data from a Brazilian cohort of AD patients, showing increased serum and plasma levels of NETs compared to age-matched controls, suggesting that complement system and NETs could be potential therapeutic targets to prevent the progression of the disease.

Finally, Ashraf et al. discussed how impaired iron metabolism contributed to oxidative stress and neurodegeneration in AD development. In detail, these authors evaluated the cerebrospinal fluid (CSF) levels of hemopexin, a heme scavenger protein, in the Alzheimer’s Disease Neuroimaging Initiative (ADNI) cohort. They found that higher CSF hemopexin levels were associated with higher CSF amyloid, preserved hippocampal metabolism, and cognitive performance. Moreover, subjects with Mild Cognitive Impairment (MCI) converting to Alzheimer’s showed higher CSF hemoglobin subunits than MCI subjects, which remained stable over time. Overall, these exciting findings indicate that iron homeostasis might be an important event in Alzheimer’s pathophysiology and a target for novel potential treatments.

Notably, the lessons from this and previous pre-clinical studies and clinical trials suggest how AD, as well as other chronic-degenerative disorders (e.g., HF, cancer) etiology is very complex and multifactorial. Thus the combination of multiple therapies is likely considered the best strategy to fight this disorder.

In conclusion, in this research topic it has been stressed the importance of novel technologies such as next-generation sequencing (NGS) because advanced the study of human diseases, identifying of novel human genome variation, and provided new potential molecular targets. Moreover, it confirmed the importance of protein-folding mechanisms in the pathogenesis of chronic degenerative disorders. Thus, further investigations of novel pathways and biomarkers represent the best scenario and opportunity to stratify patients, predict their outcome and apply specific personalized medications.
